# Determinants of Severe Hypocalcemia After Parathyroidectomy in Patients with End-Stage Kidney Disease and Renal Hyperparathyroidism: A Retrospective Cohort Study

**DOI:** 10.3390/jcm14020379

**Published:** 2025-01-09

**Authors:** Zi Kheng Tan, Wan Limm Looi, Fangxia Chen, See Cheng Yeo, Manohar Bairy

**Affiliations:** Department of Renal Medicine, Tan Tock Seng Hospital, 11 Jalan Tan Tock Seng, Singapore 608433, Singapore

**Keywords:** hungry bone syndrome, hypocalcemia, parathyroidectomy, parathyroid hormone, renal hyperparathyroidism

## Abstract

**Background**: Parathyroidectomy (PTX) is generally curative in renal hyperparathyroidism (RHPT) that is refractory to medical treatment in end-stage kidney disease (ESKD) patients. Severe hypocalcemia is a common complication of PTX and results in increased monitoring, interventions, lengths of stay, and costs of care. This study aimed to find the determinants and cutoff values of the biochemical determinants, if any, for severe post-operative hypocalcemia after PTX in adult patients with ESKD. **Methods**: Severe post-operative hypocalcemia was defined as a lowest adjusted serum calcium level < 2 mmol/L during a hospitalization stay following PTX. Receiver operating curves (ROCs) with area under the curve (AUC) values for pre-operative intact parathyroid hormone (iPTH) and pre-operative alkaline phosphatase (ALP) levels against hypocalcemia were used to determine cutoffs. Generalized linear models using Poisson regression with robust error variance were used to estimate the relative risk of severe post-operative hypocalcemia. **Results**: In total, 75 patients (38 women, 50.7%) with a mean age of 53.8 ± 11.4 years were enrolled; 43 (57%) patients developed severe hypocalcemia post-PTX and had higher pre-operative serum iPTH and ALP levels, as well as a significantly longer hospitalization post-operation (10.5 vs. 4.3 days, *p* =< 0.001). A pre-operative iPTH level of >166 pmol/L had an AUC-ROC of 0.73 and 72% sensitivity and 73% specificity, respectively, in predicting severe post-operative hypocalcemia with a relative risk of 2.00 [95% CI 1.27–3.33, *p* = 0.003]. **Conclusions**: A pre-operative iPTH level > 166 pmol/L is a strong risk predictor for post-operative severe hypocalcemia. Pre-emptive interventions in this high-risk group could potentially result in a reduced length of stay and lower acuity of care.

## 1. Introduction

Renal hyperparathyroidism (RHPT) is a common complication of chronic kidney disease. Physiological adaptation to altered levels of calcium, vitamin D, and fibroblast growth factor 23 (FGF23) leads to parathyroid gland hyperplasia and the increased production of parathyroid hormone (PTH). A sustained elevation in PTH levels, while initially compensatory, becomes pathological in due course, resulting in hypercalcemia, renal osteodystrophy, vascular calcification, and significant cardiovascular morbidity and mortality. Patients in whom medical therapy fails or those who develop complications are referred for parathyroidectomy (PTX). PTX has the potential to reduce cardiovascular and all-cause mortality, reduce symptoms and other mineral bone disease-related complications, and reduce pill burden in patients with RHPT [1]. One of the most significant and immediate post-operative complications following PTX is severe hypocalcemia owing to hungry bone syndrome (HBS).

The incidence of HBS among patients on dialysis is reportedly high, ranging from 26% to 95% [2,3,4,5]. HBS is associated with fatal hypocalcemia if not treated promptly. Despite the significant prevalence of this clinical problem, few studies have examined the risk factors for the development of HBS, and there is limited evidence on the management of calcium levels post-operatively. Managing this complication remains challenging because HBS leads to rapid, profound, and prolonged hypocalcemia, even up to three months post-operatively [6]. It is also associated with electrolyte abnormalities such as hypophosphatemia and hypomagnesemia [7].

Several authors have published protocols for managing HBS after PTX. However, these methods usually involve continuous calcium infusion and frequent blood tests. A previous study assessed the risk and severity of hypocalcemia based on alkaline phosphatase (ALP) levels and prophylactically initiated intravenous calcium replacement and found that a significant number of patients developed hypercalcemia [8]. Overall, there is a need to identify patients for targeted and personalized treatment. Setting specific cutoff values for routinely measured biochemical parameters enables clinicians to develop clinical protocols to mitigate the risks of complications. We hypothesized that it would be possible to determine cutoffs for intact parathyroid hormone (iPTH) and ALP based on what is known about the pathophysiology of RHPT and the associated biochemical derangements. Therefore, we sought to model putative risk factors for post-operative hypocalcemia and identify significant determinants and their cutoff values in our institutional cohort of patients with ESKD with treatment-resistant RHPT.

## 2. Materials and Methods

### 2.1. Inclusion and Exclusion Criteria

In this retrospective study, we screened all consecutive patients with ESKF who underwent PTX for RHPT between May 2016 and December 2022 at the Tan Tock Seng Hospital, Singapore. We excluded patients with incomplete perioperative biochemical measurements, including serum calcium, phosphate, ALP, and iPTH levels, patients with failed PTX, and patients who underwent PTX for recurrent hyperparathyroidism.

We collected data on patient demographics, including sex, age at surgery, comorbidity status, and details of dialysis, such as ESRD etiology, dialysis modality and vintage, and biochemical data, including serum levels of albumin, calcium, phosphate, iPTH, and ALP before and up to seven days after PTX, and medication use (calcium-based and non-calcium-based phosphate binders, active vitamin D analogs, and cinacalcet) from the hospital medical records. Data on the operative findings and histology of the parathyroid glands were obtained from surgical notes and pathology reports. The primary outcome of interest was severe hypocalcemia, defined as at least one adjusted serum calcium level of <2 mmol/L at any time during the post-operative period. This study was approved by the Ethics Review Board of our institution (Domain Specific Review Board of the National healthcare Group, Singapore—Ref. 2023/00169).

### 2.2. Surgical Indications and PTX

At our center, the initial management of patients with RHPT includes ensuring adequate dialysis, dietary phosphate restriction, and a combination of PTH-lowering medical therapies. Patients for whom these medical treatments fail are referred to a surgeon for the evaluation of surgical PTX, with indications including (1) persistently elevated serum iPTH level >100 pmol/L with an elevated serum ALP level, (2) uncontrolled hypercalcemia with hyperphosphatemia or clinical symptoms of RHPT refractory to medical treatment (these symptoms include calciphylaxis, bone pain, fractures, or intractable pruritus), and (3) radiological evidence of progressive metastatic calcification or features of advanced hyperparathyroid bone disease. Pre-operative imaging, such as a sestamibi parathyroid scan, is not routinely performed at our center.

### 2.3. Laboratory Data

Baseline laboratory parameters, including the serum concentration of calcium, phosphate, iPTH, and ALP before the surgery were obtained from the records for previous hospital visits or blood tests performed at the community dialysis center, whichever was closest to the surgery date and within one month prior to the PTX. iPTH levels were measured by a sandwich-type immunoassay in accredited quality-controlled laboratories. For patients on hemodialysis, the midweek predialysis iPTH was measured prior to surgery in the dialysis centers. Following surgery, iPTH levels were checked once 24 h after surgery, whereas serum calcium and phosphate levels were routinely checked at intervals of 4–6 h for the first 12–24 h. The frequency of subsequent blood tests was based on the trend in the electrolyte levels, the institutional protocol for parathyroidectomy (see Supplementary Materials File S1), and clinician discretion.

### 2.4. Medications

Baseline medication use, including phosphate binders (both calcium- and non-calcium-based), active vitamin D analogs, and cinacalcet before PTX were collected. Patients scheduled for PTX were routinely prescribed loading doses of calcium carbonate and active vitamin D analogs 3–7 days prior to the planned surgery date. Post-operatively, all patients on hemodialysis underwent dialysis using a dialysate containing 1.5 mmol/L ionized calcium. Patients on peritoneal dialysis continued on a peritoneal dialysis solution with 1.25 mmol/L ionized calcium. Initial calcium was supplemented intravenously, transitioning to oral replacement when oral intake was established with the concomitant use of active vitamin D analogs. The doses of the medications were adjusted according to the clinical symptoms and calcium level trends, aiming for a serum-adjusted calcium level between 2.15 and 2.50 mmol/L.

### 2.5. Missing Data

Pre-operative serum iPTH levels above the upper limit of the laboratory reference range (220 pmol/L) were reported as such by the laboratory (without the actual value) for two patients. The iPTH level in these patients was imputed to be 220 pmol/L as the complete case analysis method would lead to the loss of valuable data. For two patients with missing pre-operative serum ALP levels, available case analysis (pairwise deletion) was used, as they were considered to be missing completely at random.

### 2.6. Statistical Analyses

Statistical analyses were performed using Stata v14.2 (StataCorp. 2015. Stata Statistical Software: Release 14. StataCorp LLC., College Station, TX, USA). Continuous data were expressed as means ± standard deviation (SD) when normally distributed or as medians with interquartile range. We compared the demographic, clinical, and laboratory parameters between the two outcome groups using the chi-square test or Fisher’s exact test where appropriate for categorical variables. For continuous variables, *p*-values were based on the independent *t*-test or Mann–Whitney U test, where appropriate. Severe hypocalcemia was defined as at least one adjusted serum calcium level of <2 mmol/L at any time during the post-operative period. Patients were grouped into severe and non-severe hypocalcemia groups for analysis. Receiver operating characteristic (ROC) curves for PTH and ALP levels against hypocalcemia were constructed with corresponding area under ROC curve (AUC-ROC) values. In addition to the sensitivity and specificity, the positive predictive value (PPV) and negative predictive value (NPV) were calculated using Bayes’ rule with a 50% pretest probability of hypocalcemia. To identify the significant factors associated with severe hypocalcemia, significant variables from univariable logistic regression analysis and important covariates based on the previous literature were incorporated into the multivariable logistic regression model. Independent variables with cutoffs generating an AUC > 0.70 were included in the regression model as categorical variables. As the incidence of the outcome was high, the relative risk (incidence rate ratio) was estimated by generalized linear models using Poisson regression with robust error variance. The variable inflation factor (VIF) was calculated to check for multicollinearity in the linear regression model. A *p* value <0.05 was considered statistically significant.

We used the strengthening of reporting of observational studies in epidemiology (STROBE) cohort reporting guidelines [9].

## 3. Results

A total of 79 patients underwent PTX for RHPT between 2016 and 2022 at our center, and of these, 75 were included in the present study (3 patients with incomplete data for iPTH and 1 patient with recurrent PTX were excluded). The demographics of the study population and baseline parameters are listed in Table 1. Total PTX with autoimplantation was performed in 58.7% of patients, and subtotal PTX was performed in 12% of patients. Sixty-five patients (86.7%) were on hemodialysis, and nine (12%) were on peritoneal dialysis with a mean dialysis vintage of 73.4 (±32.5) months prior to PTX. Fifty-seven (76%) patients were prescribed some form of loading medication, such as calcium supplements only (4%), activated vitamin D only (16%), or both (56%), pre-operatively.

A post-operative adjusted serum calcium level <2 mmol/L was observed in 43 (57%) patients (severe hypocalcemia group), and calcium levels ≥2 mmol/L were observed in 32 patients (non-severe hypocalcemia group). Hypercalcemia (>2.60 mmol/L) was observed in 10 (13.33%) of the patients.

**Figure 1:**
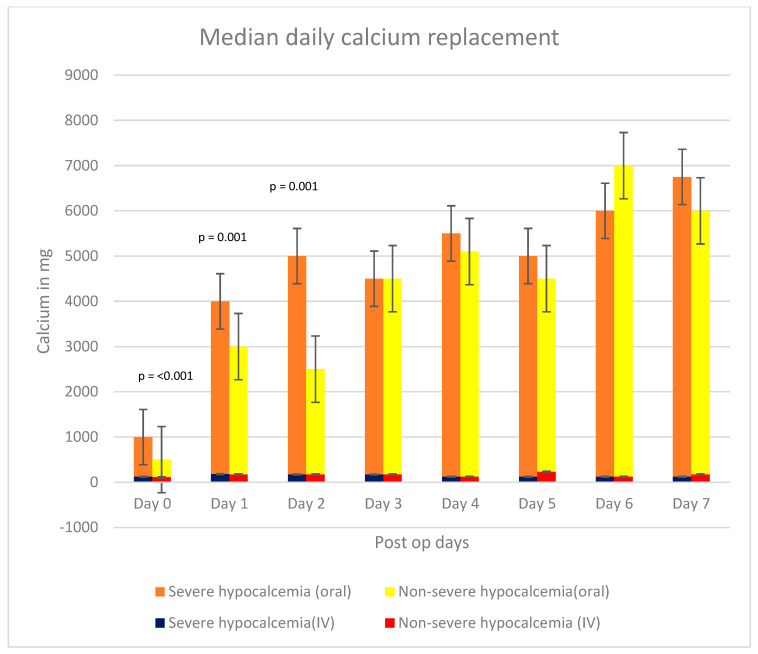
Median calcium replacement categorized by the post-operative day and severity of hypocalcemia (*p*-values for median calcium replacement from Day 3 to Day 7 were not significant).

**Figure 2:**
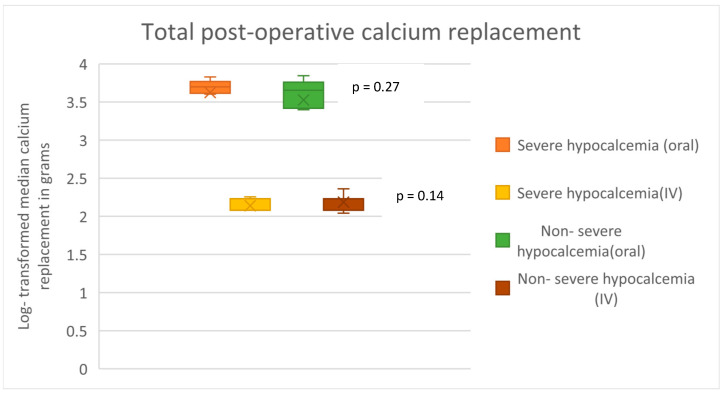
Comparison of median oral and intravenous calcium replacement doses across the post-operative period (log-transformed) in the two groups.

Patients in the severe hypocalcemia group had a significantly higher pre-operative serum ALP (380 vs. 220.5 IU/L; *p* = 0.02), iPTH level (216 vs. 129.8 pmol/L; *p* < 0.001) and a significantly longer hospital stay post-PTX (10.5 ± 1.2 days vs. 4.3 ± 0.3 days; *p* < 0.001) compared with the patients in the non-severe hypocalcemia group. Age, dialysis vintage, surgical modality, pre-operative loading medications, and pre-operative serum calcium levels were not significantly different between the two groups (Table 1). Over the 7-day post-operative period, the total amount of calcium replacement was not significantly different between the two groups, but patients in the severe hypocalcemia group received significantly higher doses of calcium supplementation during the first three days post-operation (Figure 1 and Figure 2).

**Figure 3:**
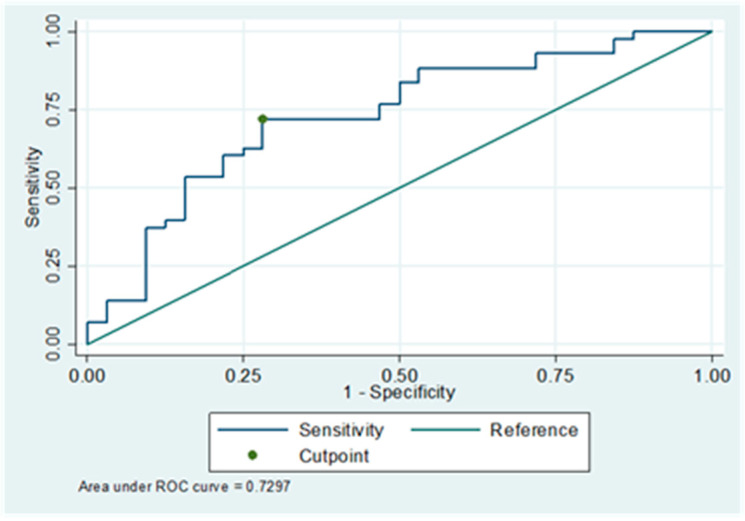
ROC curve for serum iPTH with cut–off and AUC.

**Figure 4:**
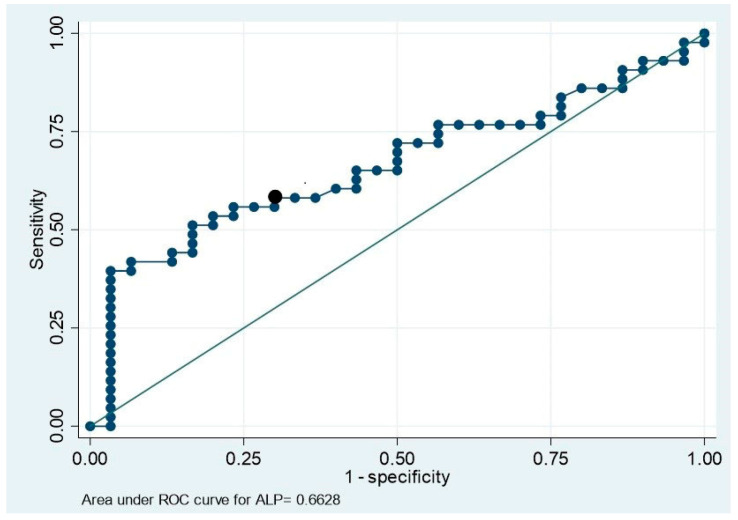
ROC curve for serum ALP with AUC.

In the univariate analysis, pre-operative iPTH and ALP levels were significantly associated with severe post-operative hypocalcemia (Table 2). ROCs with corresponding AUCs for pre-operative serum iPTH (Figure 3) and ALP levels (Figure 4) were generated, and the cutoff values were calculated. The AUC for the pre-operative serum ALP level was poor (0.67), whereas that for the iPTH level was fair (0.73) (Table 3). The PPV and NPV values for the iPTH cutoff were also higher than those for the ALP cutoff. Hence, the iPTH cutoff value was incorporated into the multivariable regression model, which showed a significant association between severe hypocalcemia post-operation and pre-operative iPTH levels above the cutoff value with a relative risk of 2.00 (95% confidence interval [CI], 1.27–1.33, *p* = 0.003) (Table 4). The mean VIF for the model was 1.15, suggesting the absence of significant multicollinearity. Other laboratory and clinical parameters, including serum ALP levels, did not show a significant relationship with the occurrence of severe hypocalcemia. A pre-operative serum iPTH level of >166 pmol/L had 72% sensitivity and 73% specificity in predicting post-operative hypocalcemia.

## 4. Discussion

Forty-three patients (57%) in our cohort developed severe hypocalcemia post-PTX. A pre-operative serum iPTH level of >166 pmol/L was the single most important predictor of severe post-operative hypocalcemia in this study. The incidence of HBS varies greatly among studies, ranging from 26% to 95% [2,3,4,5]. The vast difference in HBS incidence rates among studies is because of the lack of a universal definition for HBS and the different cutoff values being used to define hypocalcemia. HBS is usually characterized by rapid, severe, and prolonged hypocalcemia. The total serum calcium level is generally <8.4 mg/dl (2.1 mmol/L), with hypocalcemia often lasting more than four days post-operation. However, Ho et al. reported that some patients with HBS experienced substantially reduced serum calcium concentrations for up to three months post-operatively [6]. An adjusted serum calcium level of 2 mmol/L was chosen as the cutoff value to define severe hypocalcemia in our cohort. We did not observe any prolonged hypocalcemia (<2 mmol/L) for more than four days, likely because of frequent blood tests and prompt calcium replacement at our center.

Although the incidence of HBS post-PTX is high, the published studies on the management of calcium levels post-operatively are limited. Multiple risk factors have been correlated with the occurrence of HBS, including pre-operative serum calcium, phosphorus, ALP, iPTH, and albumin levels, pruritus in patients, patient age and weight, and the volume of the resected parathyroid glands. Among these, four risk factors including pre-operative serum ALP, iPTH, calcium, and age have been shown to have a stronger correlation with the occurrence of HBS post-operation [5]. Patients with higher levels of iPTH and ALP may have severe bone disease with hyperparathyroidism. In these patients, severe hypocalcemia after PTX is more likely, due to rapid bone remineralization.

Some centers use pre-operative ALP levels to guide calcium requirements after total PTX [8,10,11]. A previous study assessed the patients’ risk and severity of hypocalcemia based on ALP levels and prophylactically initiated intravenous calcium replacement over 12 h immediately post-PTX with no adjustment to the infusion, regardless of the immediate post-operative calcium or phosphate level. A significant number of patients (24.1% and 59.6% in the two groups, respectively) developed hypercalcemia in this cohort [8].

In our study, we compared patients who developed severe hypocalcemia with those who developed non-severe hypocalcemia post-operatively. Patients who developed severe hypocalcemia had higher pre-operative serum iPTH and ALP levels, as well as significantly longer hospitalization post-operation. In the univariate analysis, pre-operative serum ALP level significantly correlated with the development of severe post-operative hypocalcemia; however, it was no longer significant in the multivariable logistic regression model. Further, the AUC for the ALP cutoff was less than 0.7 with lower PPV and NPV values.

The pre-operative PTH level has also been reported to be a significant predictor of HBS and associated with higher doses of calcium replacement [12,13]. However, the iPTH cutoff value that predicts severe hypocalcemia post-operatively remains unknown. This study has identified only the pre-operative iPTH level, at a cutoff of >166 pmol/L, as a predictor of severe post-operative hypocalcemia.

We did not observe a significant difference in the incidence of severe hypocalcemia between patients who received loading calcium and vitamin D supplements and those who did not. The potentially significant association may have been masked by the relatively small sample size and lack of certainty regarding compliance with the prescribed medicines. We recommend calcium supplementation and active vitamin D analog loading prior to PTX because they pose no significant harm to patients. In a meta-analysis, Gao et al. concluded that pre-operative hypocalcemia is a risk factor for hypocalcemia after PTX [5]. In addition, pre-operative vitamin D-replete status has been shown to be associated with a reduced likelihood of severe or prolonged HBS [14,15]. We did not detect any trend towards significance, but, considering the small number of patients in our study cohort, further studies with larger sample sizes are required to confirm this association.

Throughout the six-year study period, only six patients in our cohort required intravenous calcium infusion post-PTX. Indications for IV calcium infusion included profound and persistent hypocalcemia (i.e., serum adjusted calcium concentration < 1.8 mmol/L despite intravenous bolus and oral calcium supplementation).

It is not practical to perform frequent blood tests every 4–6 h in all patients, according to the kidney disease outcomes quality initiative (KDOQI) recommendations [16]. Given the strong predictive role of pre-operative iPTH levels, we propose using pre-operative serum iPTH level > 166 pmol/L as a cutoff to identify a high-risk group of patients. Targeted intervention measures in this high-risk group may include the early withdrawal of calcimimetics before surgery, a higher loading dose of calcium supplementation, and active vitamin D analogs before surgery. These patients would also require more frequent blood tests, at intervals of 4 to 6 h after surgery, and should be empirically started on a higher dose of calcium supplementation and active vitamin D analogs immediately post-surgery to reduce the risk of potentially fatal hypocalcemia and a prolonged hospital stay.

This study has a few limitations. First, this was a retrospective observational study, and the results were limited by the usual vagaries of retrospective medical record-based studies. For example, ionic calcium, serum magnesium, intraoperative iPTH, and pre-operative vitamin D levels were not measured routinely as a standard of care in our cohort. Assays for measuring iPTH levels, despite being performed using the same method in quality-controlled laboratories, may have had slightly differing normal ranges. Second, the data were obtained from a relatively small cohort with a single-center design and thus lacked external validation. Third, owing to the heterogeneous definitions of significant post-operative hypocalcemia or HBS, we empirically decided that any serum calcium concentration < 2 mmol/L at any point during hospitalization immediately post-PTX (biochemical criterion alone, as clinical features were not captured in the data) was significant. In addition, single pre-operative levels of serum ALP and iPTH may not be representative of the degree of mineral bone disease and the risk of HBS, and bone-specific ALP was not measured. Also, although dialysis sessions in the post-operative period would have influenced serum calcium levels, this is likely to be transient in the setting of HBS and constitutes a variable that cannot be controlled as dialysis is performed for other indications with variable dialysis dose prescriptions and is inevitable in these patients.

The strengths of this study include the inclusion of almost all consecutive patients who underwent PTX for RHPT, minimal missing data, the determination of cutoff values for predictor variables, adjustment for possible confounders, and the successful identification of a predictor that can be used for developing hypocalcemia prevention protocols. In terms of generalizability, for clinicians using a severe hypocalcemia threshold of adjusted serum calcium level of 2 mmol/L, this study provides evidence for identifying high-risk groups for targeted preventive and treatment measures. We plan to conduct a prospective study incorporating the iPTH cutoff identified in this study into the protocol for post-parathyroidectomy management.

In conclusion, the early identification of the risk factors for severe hypocalcemia is important to establish intensive clinical surveillance for high-risk patients with ESKD who will benefit from aggressive medical treatment post-PTX. A pre-operative iPTH level >166 pmol/L is a strong risk predictor for post-operative severe hypocalcemia. Pre-emptive interventions to prevent this complication can result in a reduced length of stay, lower acuity of care, and significant cost savings.

## Figures and Tables

**Table 1:** Comparison of demographic and baseline laboratory data among the patient groups according to post-operative calcium level.

Variable	All Patients N = 75	Hypocalcemia Group, Adjusted Calcium <2 mmol/L N = 43	Non-Severe Hypocalcemia Group, Adjusted Calcium > or Equal to 2 mmol/L N = 32	*p* Value
Age at time of surgery in years, mean (SD), range	53.8 ± 11.4, (23–73)	52.4 ± 12.5	55.6 ± 9.7	0.24
Male, n (%)	37 (49.3)	19 (44.2)	18 (56.3)	0.30
ESKD cause, n (%)				0.57 *
CGN	33 (44.0)	19 (44.2)	14 (43.8)	
DM	24 (32.0)	14 (32.6)	10 (31.3)	
HTN	14 (18.7)	9 (20.9)	5 (15.6)	
Others	4 (5.3)	1 (2.3)	3 (9.4)	
ESKD modality, n (%)				0.37 *
HD	65 (86.7)	39 (90.7)	26 (81.3)	
PD	9 (12.0)	4 (9.3)	5 (15.6)	
Nil	1 (1.3)	0 (0)	1 (3.1)	
Dialysis duration in months, mean (SD)	73.4 ± 32.5	73.0 ± 28.4	73.9 ± 37.7	0.91
CCI score, mean (SD)	4.0 ± 1.6	3.9 ± 1.7	4.1 ± 1.5	0.68
Pre-operative serum biochemistry				
Serum albumin in g/L, mean (SD)	39.4 ± 3.6	38.8 ± 3.8	40.2 ± 3.1	0.09
Serum calcium in mmol/L, mean (SD)	2.4 ± 0.2	2.4 ± 0.2	2.4 ± 0.2	0.12
Serum phosphate in mmol/L, mean (SD)	1.8 ± 0.4	1.9 ± 0.4	1.7 ± 0.4	0.09
Serum iPTH (pmol/L), median (IQR)	169.8 (113.7, 266.7)	216 (142, 305)	129.8 (89, 186.6)	**<0.001**
Serum ALP (IU/L), median (IQR)	272 (169, 463)	380 (200, 743)	220.5 (164, 338)	**0.02**
MBD medications—long term				
Phosphate binder use, n (%)				0.17 *
Calcium-based	15 (20.0)	9 (20.9)	6 (18.8)	
Non-calcium-based	32 (42.7)	15 (34.9)	17 (53.1)	
Both	27 (36.0)	19 (44.2)	8 (25)	
Nil	1 (1.3)	0 (0)	1 (3.1)	
Type of phosphate binder—calcium-based, n (%)				0.11 *
Calcium acetate	32 (42.7)	20 (46.5)	12 (37.5)	
Calcium carbonate	10 (13.3)	8 (18.6)	2 (6.3)	
Nil	33 (44.0)	15 (34.9)	18 (56.3)	
Type of phosphate binder—non-calcium-based, n (%)				0.27 *
Lanthanum	40 (53.3)	26 (60.5)	14 (43.8)	
Sevelamer	19 (25.3)	8 (18.6)	11 (34.4)	
Nil	16 (21.3)	9 (20.9)	7 (21.9)	
Vitamin D use (Yes), n (%)	33 (44.0)	19 (44.2)	14 (43.8)	0.97
Cinacalcet use (Yes), n (%)	41 (54.7)	24 (55.8)	17 (53.1)	0.82
**Loading Medications**				
Type of loading prior surgery, n (%)				0.13 *
Calcium only	3 (4.0)	2 (4.7)	1 (3.1)	
Vitamin D only	12 (16.0)	10 (23.3)	2 (6.3)	
Both	42 (56.0)	20 (46.5)	22 (68.8)	
Nil	18 (24.0)	11 (25.6)	7 (21.9)	
Loading total vitamin D in mcg, median (IQR)	3 (0, 3)	2 (0, 3)	3 (0.5, 3)	0.65
Loading total elemental calcium in g, median (IQR)	3.8 (3, 11.3)	1.5 (0, 4.5)	4.25 (0, 4.5)	0.19
**Surgery**				
Type of parathyroid surgery, n (%)				0.71 *
Total parathyroidectomy with autoimplantation	44 (58.7)	26 (60.5)	18 (56.3)	
Total parathyroidectomy without autoimplantation	22 (29.3)	11 (25.6)	11 (34.4)	
Subtotal parathyroidectomy	9 (12.0)	6 (14)	3 (9.4)	
Length of stay following PTX (days), median (IQR), mean (SD)	5.4 (4, 9.7) 7.8 (6.8)	10.5 ± 1.2	4.3 ± 0.3	**<0.001**
Post-surgery lowest serum iPTH (pmol/L), median (IQR)	9(5, 15.7)	9.4 (6.5, 16.3)	6.8 (4.7, 14.1)	0.18

ALP, alkaline phosphatase; CCI, Charlson Comorbidity Index; CGN, chronic glomerulonephritis; DM, diabetes mellitus; ESKD, end-stage kidney disease; HTN, hypertension; HD, hemodialysis; iPTH, intact parathyroid hormone; IQR, interquartile range; MBD, mineral bone disease; PD, peritoneal dialysis; SD, standard deviation. *: *p* value for trend. Significant *p* values are in bold.

**Table 2:** Univariable logistic regression for risk factors associated with post-operative hypocalcemia.

Variable	Odds Ratio	*p* Value	Confidence Interval
Age	0.94	0.24	0.93–1.01
Female gender	1.62	0.30	0.64–4.08
Dialysis vintage(months)	0.99	0.90	0.98–1.02
Pre op serum calcium level	0.62	0.13	0.10–1.81
Loading calcium dose/day	0.94	0.25	0.82–1.05
Loading Vitamin D dose/day	0.98	0.44	0.77–1.11
No Vitamin D/Calcium loading	1.3	0.85	0.76–2.14
Pre op intact parathyroid hormone level	1.08	**0.002**	1.03–1.10
Pre op alkaline phosphatase level	1.00	**0.04**	1.00–1.02
ESKD cause (Diabetes mellitus)	1.03	0.95	0.35–2.9
ESKD on Peritoneal dialysis	0.54	0.38	0.13–2.17
Non-calcium-based phosphate binder	0.88	0.40	0.16–2.04
Total PTX without implantation	1.38	0.67	0.30–6.2

Significant *p*-values (<0.05) are in bold.

**Table 3:** ROC Cutoffs for serum iPTH and serum ALP.

	Cutoff	Sensitivity	Specificity	AUC	PPV *^,#^	NPV *^,#^
Serum iPTH (pmol/L)	166.3	0.72	0.73	0.73(fair)	72%	72%
Serum ALP (IU/L))	338.5	0.56	0.77	0.67(poor)	70%	64%

^#^ = PPV-Positive predictive value (conditional probability), NPV-Negative predictive value (conditional probability); * = Calculated using Bayes’ rule with pre-test probability of 50%.

**Table 4:** Multivariable regression model for risk factors associated with post-operative hypocalcemia.

Variable	Relative Risk *	*p*-Value	Confidence Interval
Age	0.99	0.48	0.98–1.00
Female gender	1.34	0.09	0.95–1.89
Pre-op calcium level	0.58	0.28	0.22–1.56
Pre-op ALP level	1.00	0.50	0.99–1.00
Pre-op iPTH> 166 pmol/L	2.003	0.003	1.27–3.33
Vitamin D loading dose/day	0.96	0.34	0.87–1.04
Calcium loading dose/day	0.99	0.85	0.97–1.02

Note: iPTH—intact parathyroid hormone. ALP—alkaline phosphatase. * Relative risk estimated by a generalized linear model (GLM) using the Poisson log link with robust error variance. Age, gender, serum calcium, serum ALP, loading vitamin D, and loading total elemental calcium were not significant in the above model. For continuous variables, the relative risk can be interpreted as beta coefficients.

## Data Availability

The data that support the findings of this study are not publicly available due to their containing information that could compromise the privacy of research participants but are available from the corresponding author (M.B.) upon reasonable request.

## Supplementary Materials

The following supporting information can be downloaded at: https://www.mdpi.com/article/10.3390/jcm14020379/s1, Supplementary File S1: Parathyroidectomy protocol in use during the study period with Table S1: Elemental calcium dose according to infusion rate
