# Supplementary material for: Determinants of Severe Hypocalcemia After Parathyroidectomy in Patients with End-Stage Kidney Disease and Renal Hyperparathyroidism: A Retrospective Cohort Study
Source: J Clin Med. 2025 Jan 9;14(2):379. doi: 10.3390/jcm14020379 (PMC11765800; doi:10.3390/jcm14020379)
Supplement: Supplementary file 1 [file jcm-14-00379-s001.zip › jcm-3375766-supplementary.pdf]

i. <u>Management of Parathyroidectomy in patients with ESKD</u>.

- Indications:
  - Clinical: calciphylaxis, bone pain, fractures, intractable pruritus
  - Radiological: Progressive metastatic calcification, features of advanced hyperparathyroid bone disease
  - Biochemical: Hypercalcemia without the use of Vitamin D, or with Vitamin D if Vitamin D is deemed necessary for control of secondary hyperparathyroidism. Calcium x phosphate product > 6, with persistently elevated ALP. Persistently elevated iPTH > 100 pmol/L with no response to Vitamin D or unable to give Vitamin D in adequate dosage.
- Pre-Operative Preparation:
  - A Sesta-MIBI parathyroid scan should be ordered for the patient, if the patient is a candidate for surgical parathyroidectomy. After which, the patient should be referred to the endocrine surgeon for resection of the parathyroids.
  - The patient should be informed to contact the primary nephrologist of the date of the surgery, as the patient should be started on calcium carbonate and Vitamin D prior to the surgery, so as to reduce the occurrence of hungry bone syndrome. Commonly they are prescribed calcium carbonate 1.25g TDS, and Calcitriol 1-2 mcg OM 3 days before the surgery.
  - If the serum ALP > 500 U/L, inform the Anesthetist to insert a central venous line during the surgery, in anticipation for continuous intravenous calcium infusion.
- Post-Operative Management – General Principles:
  - The target range for albumin-corrected serum calcium is 2.1 to 2.5 mmol/L. Patients who have undergone subtotal parathyroidectomy are likely to have lower requirements for calcium and Vitamin D than those who have had all four glands removed.
  - Check serum calcium, phosphate and albumin within 4 to 6 hours of surgery, and every 6 to 12 hours thereafter. The frequency of monitoring should be determined by both the absolute level and rate of change of calcium levels. Daily monitoring can be instituted after 48 hours, assuming that serum calcium levels are > 2.1 mmol/L.
  - Administer either oral or intravenous calcium according to serum calcium levels: if serum calcium is > 1.8 mmol/L, give oral calcium supplements; if serum calcium is < 1.8 mmol/L or is falling rapidly (e.g. >10% over 4 to 6 hours) OR the patient is symptomatic, give intravenous calcium replacement.
  - Continue Calcitriol 1-4 mcg OM, but dose should be reduced if serum calcium levels > 2.4 mmol/L.
  - Continue usual non-calcium based phosphate binders, prescribed with meals, unless there is hypophosphatemia.
  - Monitor iPTH once, 24 hours after surgery.

- Post-Operative Management – Oral Calcium Supplements:
    - o Oral calcium supplements should be prescribed to be taken between meals, to maximize absorption.
    - o Prescribe calcium carbonate 1.25g TDS, increasing to 3.75g QDS according to serum calcium levels and response.
- Post-Operative Management – Intravenous Calcium:
    - o A 10ml ampoule of 10% Calcium Gluconate contains 90mg (2.2 mmol) of elemental calcium.
    - o Intravenous calcium may cause hypotension and injection site reactions, and it is advised that injection is given via a large peripheral or central vein.
    - o In an emergency situation, administer 10 to 30 mls of 10% calcium gluconate intravenously as a slow bolus (over 10 to 15 minutes, with a maximum rate of 0.44 mmol of calcium per minute). Continuous ECG monitoring should be instituted.
    - o In other situations, calcium can be administered as a continuous infusion. Prepare 100ml of 10% Calcium Gluconate in 150ml of 5% Dextrose (to a total volume of 250ml). Start at an initial rate of 20 to 40 mls/hour (equivalent to 1 to 2 mg/kg/hour of elemental calcium, assuming a body mass of 70kg).
    - o Adjust the rate of infusion according to the response (i.e. serum calcium measured 6 hours later): increase rate by 25% to 50% if serum calcium < 1.8 mmol/L; continue at same rate if serum calcium is 1.8 to 2.1 mmol/L; decrease and stop if serum calcium > 2.1 mmol/L.
    - o The infusion rate should also take into account the amount of oral calcium that the patient is receiving, and the aim should be to provide adequate oral calcium replacement at the earliest opportunity.
    - o The table below indicates hourly and daily elemental calcium doses according to the infusion rate:

Table S1. Elemental calcium dose according to infusion rate

Rate of Infusion (ml/hr)	Dose of Elemental Calcium	
	Hourly Dose (mg)	Daily Dose (g)
20	72	1.7
40	144	3.5
60	216	5.2
80	288	6.9

- Post-Discharge Management:
    - o Requirements for calcium supplements and calcitriol upon discharge are likely to vary considerably between individual patients, and advice should be sought from the Renal Consultants regarding the exact doses and follow-up arrangements.
      In general, check the serum calcium, phosphate and albumin levels daily for the first week after surgery until the levels are stable. After discharge, check the levels twice a week for the first 1-2 weeks and then weekly until the levels are stable.
